# Evaluating the Impact of a Pragmatic Nutrition Awareness Program for Expectant Mothers upon Birth Weight of the Newborn

**DOI:** 10.1093/ecam/neq034

**Published:** 2011-03-09

**Authors:** Sanjeev Rastogi, Ranjana Rastogi, Devesh Rastogi, Rajiv Rastogi, Girish Singh, Francesco Chiappelli

**Affiliations:** ^1^Department of Kaya Chikitsa, State Ayurvedic College and Hospital, Tulsi Das Marg, Lucknow 226004, India; ^2^Department of Obstetrics, Vatsala Hospital, Lucknow, India; ^3^Indulgence Food (Pvt.) Ltd, Nagpur, India; ^4^Central Council for Research in Yoga and Naturopathy, New Delhi, India; ^5^Department of Community Medicine, Banaras Hindu University, Varanasi, India; ^6^UCLA School of Dentistry, Division of Oral Biology and Medicine and Associated Clinical Specialties, Los Angeles, CA, USA

## Abstract

Poor maternal nutritional status and substandard antenatal care, which result in increased women's risk, low birth weight and stillbirth, afflict many countries with weak or emerging economies even today. Studies that address the effect of extending nutrition awareness among pregnant women to the net outcome of pregnancy remain scarce. We aimed to compare and contrast the effect of a pragmatic nutrition awareness program for expectant mothers (NAPEM) on birth weight of the newborn with a control group who received no such nutrition awareness activity. The effect of variables of mode of newborn delivery, associated complications at birth, and APGAR score of the newborn were also assessed. A pragmatic intervention trial of an antenatal care (ANC) program that consisted in nutrition awareness was conducted involving 53 pregnant women. Awareness was given through one-to-one interview and through informational literature provided to the participants in the local language. A hospital registry for deliveries undertaken during the study period was screened for identification of variables. A control group of matched pregnant women (*n* = 53) was obtained from the same hospital registry from preceding years, when the nutrition awareness program was not executed. A statistically significant improvement in birth weight of the newborn was observed in the intervention group, where expectant mothers were made aware about desired nutrition during pregnancy. A reduced incidence of complications associated with pregnancy was also observed in the intervention group. Providing awareness about nutritional requirements during pregnancy and suggesting the pragmatic ways to meet them was shown to be one possible effective measure to deal with pregnancy-related undernutrition. We show the efficacy of the intervention for underprivileged regions of India marked by inadequate health care delivery and lower socio-economical standards. We discuss our findings in the context of available evidence-based guidelines.

## 1. Introduction

Improving the health of pregnant women is critical to their prospects of surviving pregnancy and childbirth and to their long-term health. It is also pivotal to improving the survival and health of children [1]. It follows that the nutritional status of the expectant mother is among the most important determinants affecting pregnancy outcomes, including the birth weight of the newborn [2, 3]. In order to support the growing fetus and also to prepare the expectant mother for the upcoming events of childbirth and subsequent feeding, pregnancy leads to a plethora of physiological changes in the mother that converge at ensuring an efficient handling of the requisites emerging subsequent to pregnancy. In brief, a qualitative and a quantitative modification of nutrients intake is essential for a pregnant woman. This modified requirement of nutrients during pregnancy can either be met through nutrient supplementations or through a qualitative modification of food intake to ensure the required supply.

In developing countries, maternal undernutrition is a significant cause of pregnancy-related poor outcomes including maternal mortality, low birth weight and stillbirth [4]. India, for example, reports a 28% low birth weight prevalence, which is significantly higher than its global occurrence [5]. Pregnancy-related undernutrition in developing countries can be attributed to various socio-economical reasons. In addition, poor awareness of basic nutritional requirements during pregnancy and practical and economical ways to meet these requirements further contributes to the problem [6].

Iron and folate distribution to expectant mothers through primary healthcare centers is the usual method of nutrition interventions during pregnancy in India. However, this distribution is limited to those women who are availing an antenatal care (ANC) at a health care facility. In India, a regular ANC at a healthcare facility is poorly delivered and largely unsuccessful, particularly in rural areas where the healthcare delivery system is scarce making the situation gloomier. The National family health survey (NFHS)-3 of India indicates that only 77% pregnant women have at least one ANC at a healthcare facility during their pregnancy. In certain States in India, where gross inequality of healthcare exists, this percentage can drop as low as 34%. A poor distribution of iron and folic acid among those receiving ANC (only 65% receive such a supplementation) and an additional poor compliance to supplements (only 23% take the supplementation for recommended 90 days) further contributes to poor nutrition during pregnancy in India [4]. National Rural Health Mission (NRHM), which was launched in India in 2005 with an objective of dealing with various regional issues related to unequal health care delivery specially to maternal and child health care segment, so far could not create a noticeable impact upon the overall health status of the marginalized population [7]. Taken together, these data indicate that a nutrient supplementation to pregnant women through healthcare facilities may not be the most dependable way to deal with pregnancy-related undernutrition in India.

**Figure 1:**
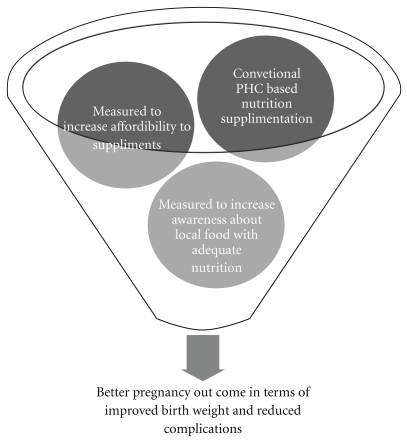
A better pregnancy outcome proposal through combining conventional ANC and local dietary customs.

Taken together, these findings suggested an alternative approach to deal with pregnancy-related undernutrition. Specifically, the purpose of our study was to test whether or not an effort to increase awareness about nutrition requirements during pregnancy could help significantly in improving the nutrition status among pregnant women in terms of birth weight of the infant. It was hypothesized that extending awareness about actual nutrition requirements during pregnancy and suggesting their pragmatic solutions would eventually lead to a better self-care among pregnant women (Figure 1). We outlined a diet-based nutritional plan for pregnant women supplemented with traditional herbal adjuvant supposed to help an uneventful delivery and child wellbeing. Herbal medicine is described as the use of plant materials in medicine and food for therapeutic purposes. Various herbal remedies are used during the prenatal period to “prepare" the uterus and cervix for childbirth and ease pain during labor and delivery [8]. The attitude of pregnant women toward CAM, particularly with regard to complementary and alternative drugs (CADs), including herbal drugs and other products of natural origin, seems to be an appealing approach to guarantee the well-being of their unborn children. In a recent report, Lapi et al. [9] found 48% of pregnant women from Italy taking at least one CAD previously and during the current pregnancy. Consistently in the last decade, an important increase in the use of CAM has been observed in Europe, USA and Australia. Most of these studies showed women as the major users of alternative medicines when compared with men [10–12]. This attitude of women toward herbs including food was proposed to be a positive factor supporting increased compliance for any food-based intervention meant to propose a safer pregnancy outcome.

In support of our hypothesis, a Cochrane systematic review had identified five studies upon 1134 pregnant women provided with advice for an improved protein and energy intake as the only intervention during pregnancy. The effects were analyzed in terms of nutritional status and net pregnancy outcomes. Overall, the reported evidence-based findings showed significant positive changes in the mothers' nutritional status, but inconsistent outcomes with respect to net pregnancy outcomes [13].

In a translational mode, we crafted a novel intervention program based on these evidence-based results, which provided greater awareness about practical, economical and locally customized approaches to meet the nutrition requirement during pregnancy. We pilot-tested this pragmatic Nutrition Awareness Program for Expectant Mothers (NAPEM) in the pragmatic clinical trial reported here. We evaluated the outcome of NAPEM by contrasting birth weight of the newborn among the expectant mothers in the intervention group, comparing them with a group of matched control infants born of mothers at the same healthcare facility, who did not undergo NAPEM. We discuss our findings in the context of recent systematic reviews, which, taken together, provide compelling grounds for newly revised evidence-based clinical practice guidelines.

## 2. Methods

### 2.1. Study Design and Measures

A pragmatic trial was designed to explore the effects of an indigenized NAPEM. The NAPEM program contained two key elements. First, it provided awareness regarding actual nutrition requirements during pregnancy. Second, it proposed a practical and an economical approach that was specific and appropriate to the indigenous culture, site and locale, and that aimed specifically to meet the nutritional requirements during pregnancy. This component of NAPEM also contained specific inputs to assure optimal availability of the desired nutrients during pregnancy through appropriate food combinations, cooking and intake methods.

To ensure proper delivery of the NAPEM intervention, an active program was devised that included extending one-to-one interaction with all the expectant mothers receiving ANC at a healthcare facility. The participants were provided with a supplementary take-home brochure containing the key messages designed in their local language. These dietary instructions were supplementary to conventional ANC recommendations at the healthcare facility, which included the medications recommended for the ANC.

Following launch of NAPEM, initial intervention was provided to every pregnant woman who had attended the outdoor care at the healthcare facility. Actual outcome was measured by observing the hospital registry for maternity services after 3 months of launch of the NAPEM program and continued for 3 months thereafter.

Birth weight of the newborn was used as the key outcome variable for evaluating impact of the program, however the mode of delivery be, associated complications during pregnancy or at the time of delivery; the APGAR scores of the newborns were also recorded.

Expectant mothers fulfilling the inclusion criteria (*n* = 53) were evaluated, and compared with control pregnant women (*n* = 53). The control group was identified through the hospital registry for a corresponding period in the preceding year when no such nutrition awareness activity was observed in the hospital. In order to match the women in the intervention and the control groups, subjects were assigned to the control group as they were receiving the similar ANC from same hospital but were devoid of the gains of any additional nutrition awareness program. The program was designed in adherence to ethical standards, and an approval of ethics research committee was obtained. Informed written consent from patients participating in active intervention was obtained prior to their participation in the study.

### 2.2. Setting

The study was conducted at Vatsala Hospital, a Merry Gold Health Network (MGHN) accredited level 2 maternity healthcare facility at Lucknow, India. NAPEM was launched on February 1, 2009. The hospital registry for deliveries conducted from May 1, 2009 to July 30, 2009 was screened for study. Age of the mother, mode of delivery, associated medical illness, complication occurred during delivery, APGAR score of the newborn 1 min after birth, and birth weight of the newborn were extracted from the hospital registry. Similar data were collected through screening of the hospital registry for deliveries conducted in corresponding period of the preceding year for the control subjects.

### 2.3. Patient Selection

Subjects for the intervention and the control groups were selected by screening the hospital registry for deliveries undertaken during a prescribed period. Only term deliveries with records of regular ANC from the same hospital were included. Subjects fulfilling these criteria were included irrespective of age, parity or outcome of the preceding delivery.

Attending pediatrician's note for determination of term or preterm babies was used to differentiate term from preterm deliveries. Subjects who were receiving ANC from the hospital were identified by their registration cards.

### 2.4. Statistical Analysis

Mode of delivery was expressed as a categorical variable, and analyzed by chi-square analysis (Fisher Exact test for 2 × 2 design). Descriptive statistics (mean ± standard deviation (SD)) were obtained for birth weight. Assumptions for parametric statistics were tested and met, and the means compared by unpaired, equal sample size *t*-test. The level of significance was taken at alpha = 5%.

Descriptive statistics (i.e., percentage) were obtained for other outcome variables, including complications during pregnancy and delivery and APGAR score.

## 3. Results

A screening of hospital registry from May 2009 to July 2009 found 53 deliveries fulfilling the inclusion criteria as determined for study. These subjects, exposed to the NAPEM intervention, were recorded for age of mother, mode of delivery and birth weight of the newborn through the hospital records. Correspondingly, control expectant mothers (*n* = 53), presented with the customary ANC but not including the NAPEM intervention under test here, and otherwise fulfilling the eligibility criteria, were taken up from the hospital records of the corresponding period in the preceding year.

### 3.1. Age of the Participants

The average age of the participants of intervention group was 25.5 years and 24.4 years in the control group. A comparison of age distribution in the two groups was not statistically different.

### 3.2. Mode of Delivery

**Figure 2:**
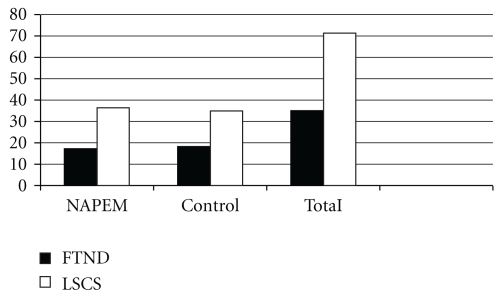
Mode of delivery in intervention and control groups.

No significant difference was observed in the ratio of mode of delivery between two groups; it approximated for a 1 : 2 ratio for full-term normal delivery (FTND) and lower segment Cesarean section (LSCS) in both the groups (Table 1, Figure 2).

### 3.3. Birth Weight of the New Born

The NAPEM intervention did reveal a significant difference (*P* <  .05) in birth weight of the newborn, when compared with the control group. The mean birth weight of newborn in the NAPEM intervention groups (mean weight in kg ± SD: 3.067 ± 0.46) was significantly higher than that of the control group (2.860 ± 0.45) (Table 2). The birth weight of NAPEM newborns was, on an average, found *∼*207 g (7.24%) higher in the NAPEM intervention group, when compared with the birth weight of infants in the control group.

### 3.4. Associated Complications during Antenatal Period and during Delivery

**Figure 3:**
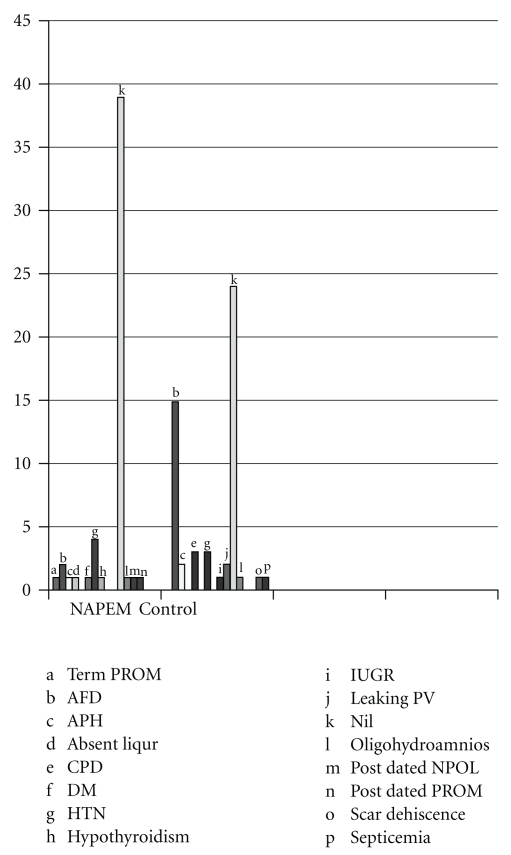
Pre- and post-natal complications in control and intervention group.

The data also suggested a trend toward a reduced incidence of complications associated with pregnancy or delivery in the NAPEM intervention group (uneventful delivery: 73% in intervention group versus 45% in control group (Table 3, Figure 3), as well as differences in the APGAR score (Table 4). Neither sets of differences attained statistical significance due to the small sample size in this pilot trial.

## 4. Discussion

We report preliminary outcomes of a pilot pragmatic trial crafted on the basis of the best available evidence generated from the process of research synthesis of nutritional status and pregnancy outcome. The Cochrane systematic review [13] established the need for further investigation of the effects of improved awareness of prenatal nutritional status and net pregnancy outcomes. Therefore, and in translational mode of evidence-based medicine, we developed the novel NAPEM intervention, which we tested in a pilot pragmatic trial.

NAPEM was directed at providing awareness about prenatal nutrition requirements, and at articulating a creative approach for the site- and language-specific dissemination in order to ensure optimal delivery of nutritional supplementations by means of appropriate indigenous foods, cooking and other intake methods (e.g., drinking).

NAPEM, a first of its kind in terms of both, the fact that it is grounded on evidence-based outcomes and that it addresses culture-specific adoption and intake processes, which are complementary to those ANC traditionally utilized in Western medicine, demonstrated a clear and statistically significant improvement in birth weight of the newborn, when compared with the infant birth weight from expectant mothers who had undergone the traditional ANC, devoid of NAPEM. It is noteworthy, moreover, that the mean birth weight of infants born to mothers in the intervention group (ANC supplemented with NAPEM) (3.067 ± 0.46) was greater than the Indian normal mean birth weight (2.700–2.800 kg) [6], which itself was consistent with the birth weight recorded in the control group (2.860 ± 0.45 kg).

That no difference was uncovered in terms of modes of delivery (i.e., FTND versus LSCS) between the groups suggests that a maternal nutrition has little effect upon the mode of delivery performed at a healthcare facility. A reduced incidence of complications during pregnancy in the intervention group further suggests a better positioning of mothers for events of delivery if they are better nourished.

The outcomes of the pilot pragmatic trial study presented here suggest a significant value to the nutrition awareness campaign as a mode to intervene in pregnancy-related undernutrition, specifically in terms of improving infant birth weight. Nevertheless, a potential bias from the study may lie in the form of improper selection of the subjects. The two groups of subjects under the study were not evaluated for their corresponding weight gains during the pregnancy period, which could have been taken as a direct measure to observe the impact of nutrition awareness upon the recipients. Furthermore, a nutritional status of the expectant mother at the time of their registration was also not accounted in the study. These threats to the internal validity of our reported observations are compounded by the fact that our sample was obtained from one healthcare facility in India, which limits the external validity of the findings.

Parity of the mother is also found to play a significant role in pregnancy-related care among expectant mothers. A better ANC compliance is reported among women with a higher parity [4]. It was not reported in the undertaken study. Participants were also not observed for their compliance to the suggestions made about their nutritional requirements.

Nonetheless, the results of this pilot pragmatic trial strongly suggest that a well-crafted NAPEM intervention can substantially improve perinatal outcome, specifically in terms of infant birth weight. A larger sample size will be needed to establish the statistical significance of other related compelling trends in the data.

Despite the limitations of this pilot trial, the study is critical and timely, and provides a significant inference about potential use of extending nutrition awareness as a prospective tool to intervene in pregnancy-related undernutrition. An improved birth weight of the newborn is one of the strongest determinants, which signifies the future prospects of the infant in terms of their survival [14]. A recent study has shown promises of a prenatal home-visitation program with focus on social support, health education and access to services for reducing low birth weight deliveries among at-risk women and adolescents [15]. This study can be of special significance to India where undernutrition during pregnancy is still a potential cause of pregnancy-related complications and maternal mortality besides its direct impact upon weight and subsequent survival of the newborn.

Considering the unabated and unequivocal importance of nutrition during pregnancy and considering the inadequate access to nutritional requirements among Indian women, primarily because of unavailability and secondarily because of lack of awareness, it is important and urgent to establish the foundations of a nutrition program designed to educate women for the possible ways to meet out their requirements through simple modifications of conventional food and also through simple knowledge of certain conventional foods more apt for pregnancy. Incidentally, the knowledge of immuno-modulating effects of food and also the therapeutic values of certain foods, which can also be useful for various pregnancy associated ailments, further underscores the importance of food-related interventions during pregnancy [16, 17]. As we need truly customized interventions conformingto various factors such as life style, age and physiology-related conditions, we further need to look at natural and more sustainable opportunities to give a living touch to interventions [18]. NAPEM is one example of an intervention program that adds to the nutritional value of food without adding much to its cost, which would be highly desirable in developing countries where the average household unit does not have a liberty to expand its monthly budget to seek specialized foods for special physiological conditions.

In this study, we have proposed NAPEM as a simple nutrition awareness program among pregnant women in a North Indian urban setting. In this paradigm, expectant mothers were made aware of their nutritional requirements during pregnancy, and also of simple domestic measures that could be considered to overcome these nutritional gaps. The NAPEM intervention was evaluated for its clinical outcomes in terms of birth weight, and led to a clear and statistically significant improvement in birth weight of the newborn.

Improving the birth weight of newborn and consequently reducing the incidence of low birth weight lowers infant mortality rates and has multiple additional benefits. A recent study estimates the economic gains of reducing the incidence of low birth weight in low-income countries through lower mortality rates and medical costs and through increased learning and subsequent productivity. The estimated economic benefits, under plausible assumptions, are presumed to be fairly substantial. Interventions aiming to improve the birth weight are also warranted for developing countries on the grounds of resource saving and increasing productivity [19].

The State of Asia Pacific Children (SOAPC) 2008 report of UNICEF clearly identifies Indian role in global attainment of the health-related millennium development goals. India's achievements in improving health, nutrition, water and sanitation, education and child protection, gender equality and women's empowerment in the coming years are considered crucial to balance the global health markers [20]. The pragmatic nutrition awareness intervention among expectant mothers we have tested here in one part of India that is marked by poor maternal and child health standards points to further exploration of novel ethnic- and language-specific approaches to expedite smooth transition for meeting India's health-related millennium development goals sooner.

Translational evidence-based medicine must draw novel clinical trial interventions from the best available evidence derived from systematic reviews, as we have done in this study. But, it must as well rejoin, with the findings it obtains, the current state of research synthesis in order to contribute to the formulation and testing of revised and recommended clinical practice guidelines. In this light, it is informative to place our findings, obtained in a hospital setting in India, in the context of evidence-based policies being currently formulated. For example, and based on the best available evidence, it is the opinion of the American Dietetic Association that women of child-bearing ages and pregnant women should maintain good nutritional status through a lifestyle to optimize maternal health and to reduce the risk of birth defects, suboptimal fetal growth and development, low birth weight and chronic health problems in their children [21]. Case in point, a recent systematic review by the World Health Organization has concluded that “universal prenatal supplementation” with iron or iron and folic acid given during pregnancy either daily or weekly should be recommended to prevent anemia and iron deficiency at term. The best available evidence failed, however, to indicate that such supplementation would significantly reduce maternal as well as neonatal adverse clinical outcomes, including low birth weight, delayed development, preterm birth, infection or postpartum hemorrhage [22]. Specifically, the best available concerted research evidence indicates that a significant reduction in the risk of low birth weight is obtained among infants born to women who received multi-micronutrients during pregnancy, compared with placebo (relative risk (RR) 0.81, 95% confidence interval (CI): 0.73–0.91) or iron-folic acid supplementation (RR 0.83, 95% CI: 0.74–0.93). Birth weight was significantly higher among infants whose mothers were in the multi-micronutrient group than among those whose mothers received iron-folic acid supplementation (weighted mean difference 54 g, 95% CI: 36–72 g) [23]. These data fully confirm the results of the present pragmatic clinical trial conducted within the developing healthcare system of an emerging economy, such as India.

The findings of the pilot pragmatic trial we report here are particularly important because a cross-national perspective of evidence-based medicine on prenatal care indicates that ANC, while widely used in low- and middle-income countries because it provides a natural facility-based contact through which to provide or educate about many of the interventions, generally yields no or little clear evidence of benefit from a programmatic perspective. Therefore, the vast majority of global stillbirths still occur in countries with weak or emerging economies, because poor nutritional status, lack of ANC and a number of behaviors increase women's risk of low birth weight and stillbirth in many resource-poor settings. Evidence-based recommendations for interventions to reduce these risks have been proposed with the goal to reduce the resulting burden of low birth weight and stillbirths [24]. The evidence for the beneficial impact of such interventions has not yet been comprehensively evaluated until this pragmatic trial we have reported here.

Referring to the proposal of optimal ANC particular to India, this would be contextual to find a good nutrition-based ANC description in Ayurvedic classics. In recognition to temporal changes in growing fetus and changing nutrition requirement to fetus and subsequently to mother, Ayurveda proposes a monthly dietary regimen to ensure an optimal growth of fetus besides preparing mothers for the upcoming event of child birth. This is important to observe here that these regimen, as recommended during pregnancy, are essentially composed of various food combinations in addition to herbs having feto-protective and promotive actions. Many of these recommendations of Ayurveda providing traditional care to expectant mother are still in vogue as folklore [25, 26].

## Figures and Tables

**Table 1:** Mode of delivery according to group.

Mode of delivery	Group		
	NAPEM intervention *N* (%)	Control *N* (%)	Total *N* (%)

FTND	17 (32)	18 (34)	35 (33)
LSCS	36 (68)	35 (67)	71 (66)

Chi-square = 0.043, *P* >  .05 NS.

**Table 2:** Infant birth weight (Kg).

	Group	
	NAPEM intervention (*n* = 53)	Control (*n* = 53)

Mean (SD)	3.0672 (0.4603)	2.8604 (0.4525)

Unpaired, equal sample size *t* = 2.332, *P* <  .05.

**Table 3:** Pre- and peri-natal complications.

Complication	Group		
	NAPEM intervention *N* (%)	Control *N* (%)	Total *N* (%)

Term PROM	1 (1.90)	0 (0.00)	1 (0.90)
AFD	2 (3.80)	15 (28.30)	17 (16.00)
APH	1 (1.90)	2 (3.80)	3 (2.80)
Absent liquor	1 (1.90)	0 (0.00)	1 (0.90)
CPD	0 (0.00)	3 (5.70)	3 (2.80)
DM	1 (1.90)	0 (0.00)	1 (0.90)
HTN	4 (7.50)	3 (5.70)	7 (6.60)
Hypothyroidism	1 (1.90)	0 (0.00)	1 (0.90)
IUGR	0 (0.00)	1 (1.90)	1 (0.90)
Leaking PV	0 (0.00)	2 (3.80)	2 (1.90)
Nil	39 (73.60)	24 (45.30)	63 (59.40)
Oligohydroamnios	1 (1.90)	1 (1.90)	2 (1.90)
Post dated NPOL	1 (1.90)	0 (0.00)	1 (0.90)
Post dated PROM	1 (1.90)	0 (0.00)	1 (0.90)
Scar dehiscence	0 (0.00)	1 (1.90)	1 (1.90)
Septicaemia	0 (0.00)	1 (1.90)	1 (1.90)

Total	53 (100)	53 (100)	106 (100)

**Table 4:** APGAR score.

APGAR	Group		
	NAPEM intervention *N* (%)	Control *N* (%)	Total *N* (%)

10 = 10	6 (11.30)	0 (0.00)	6 (5.70)
6 = 10	1 (1.90)	1 (1.90)	2 (1.90)
7 = 10	4 (7.50)	0 (0.00)	4 (3.80)
8 = 10	42 (79.20)	52 (98.10)	94 (88.70)

Total	53 (100)	53 (100)	106 (100)
